# Models to define the stages of articular cartilage degradation in osteoarthritis development

**DOI:** 10.1111/iep.12230

**Published:** 2017-06-05

**Authors:** Blandine Poulet

**Affiliations:** ^1^ Institute of Ageing and Chronic Disease University of Liverpool Liverpool UK

**Keywords:** cartilage, mouse models, osteoarthritis

## Abstract

Osteoarthritis (OA) is a common chronic disorder that affects an increasing number of the ageing population. Despite the prevalence, there are currently no therapies. Defining new therapies that target specific pathogenic phases of disease development relies on the effective separation of the different stages of OA. This manuscript reviews the tissues and models that are being used to separate these stages of disease, in particular initiation and early and late progression. These models include human tissues with known initiating factors, the use of anatomical locations with defined relationships to the primary cartilage lesion area, timing of OA development in well‐described animal models and the versatility of a non‐invasive model of murine knee joint trauma.

## Introduction

**Figure 1:**
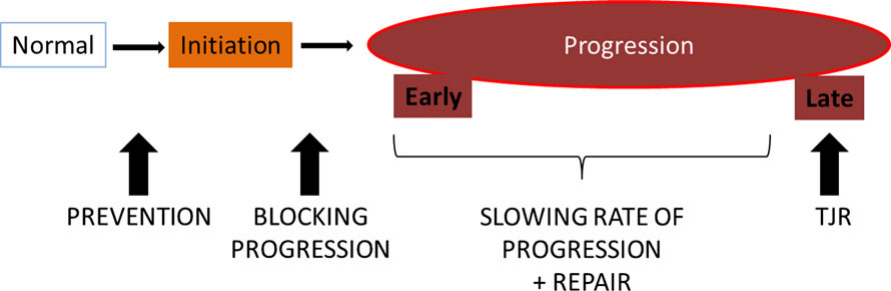
Stages of osteoarthritis and potential target points for therapy. Prevention therapy can be used before disease initiation in normal joints; blocking progression from taking place may be targeted through events that make joint become progressively worse with time. During progression, early and late stages should be separated as slowing of the rate of progression and repair potentials will be more successful in the earlier stages of disease, whereas total joint replacement (TJR) may be the only option left for late stages of disease.

Osteoarthritis (OA) is the most common degenerative disease of synovial joints affecting more than 6.6 million people in England alone. Despite this high prevalence, there are currently no effective therapies for patients with osteoarthritis. Determining new targets for therapy, for both prevention and slowing of disease, relies on defining the different stages occurring during osteoarthritis development. These involve initiation, early and late stages of progression, each of which can conceivably be targeted selectively to delay the need for joint replacement surgery (Figure 1).

A number of risk factors are known to be involved in OA, including genetics, mechanical instability and joint injuries, ageing, obesity. OA development often relies on the interactions between various factors, making it difficult to pinpoint which mechanism should be altered to slow disease progression, or to prevent disease initiation in specific populations. In addition, the idea that a treatment should be linked to initiating factor is plausible: some studies in mouse models have now shown that specific treatment/mechanisms can modify ageing‐associated and trauma/mechanical‐induced OA differently (Little & Zaki 2012; Rowe *et al*. 2017; Usmani *et al*. 2016). Hallmarks of OA include articular cartilage (AC) degradation, with loss of proteoglycan and collagen type II, two of the most prevalent components of cartilage, subchondral bone sclerosis, osteophyte formation, and synovial hyperplasia and activation. The sequence of these changes (i.e. do changes start in the cartilage or in the bone?) is still disputed (Brandt *et al*. 2006; Little & Fosang 2010) and will be dependent on the patients and models used for research. Indeed, it has been shown that overexpression of EphB4 specifically to bone protects from OA development and AC degradation (Valverde‐Franco *et al*. 2012). In contrast, global and cartilage‐specific (Col2a1) deletions of MMP13 both protect against post‐traumatic OA (Little *et al*. 2009)(Wang *et al*. 2013)), suggesting in this case MMP13 affects cartilage directly.

Determining stages of diseases has important implication for therapeutic strategies, as potential targets may affect initiation, early and late stages of diseases differently. A recent review on standardizing OA definition highlights the need to stage disease, separating OA development, OA progression and early detection (Kraus *et al*. 2015). In addition, it was recently found different outcomes between short‐and long‐term inhibition of CCR2, suggesting this chemokine may play different roles during OA progression (Longobardi *et al*. 2016). For example, is the beneficial effect of a specific treatment due to a protection against initiation of AC lesions, or because of interference with a specific event involved in progression of joint degeneration? Furthermore, separating the different phases of OA development is likely to help in the search for markers of disease and allow for appropriate targeting of patients that are most likely to respond to specific treatment. What separates these stages and how to define them clinically is still largely unknown, although it is now well accepted that some disease stages show different cellular processes (Anderson *et al*. 2011; Jiang & Tuan 2015; Liu‐Bryan & Terkeltaub 2015). This review summarizes recent approaches to differentiate the stages of OA progression in both human and animal studies.

## Defining the initiating event in human patients

A major issue with separating disease stages lies with the determination of the initiating factor. Although many risk factors for osteoarthritis development are well known, including ageing, genetics, obesity and mechanics, it is often impossible to determine the initiating event. Determining the initiating factors in many patients with osteoarthritis challenging as they present themselves at advanced stages of the disease; however, this might be possible in small subsets of patients. These small groups may then benefit from targeted therapies in the earlier stages of disease.

One such group is represented by those who have suffered from a severe joint injury. These are usually well monitored, with regular follow‐up, in particular at the earliest stages after the trauma. Specific examples include intra‐articular fractures, ligament and meniscal injuries. (For further detail of post‐traumatic OA and their animal models currently used, see recent reviews: Lohmander *et al*. (2007), Christiansen *et al*. (2015)).

Another possible group is linked to genetic predisposition to osteoarthritis. These specific genetic mutations that ‘guarantee’ OA development may include those that affect cartilage matrix genes, which are also associated with early‐onset osteoarthritis and joint chondrodysplasias (Hecht *et al*. 1995; Muragaki *et al*. 1996; Paassilta *et al*. 1999; Gleghorn *et al*. 2005; Rukavina *et al*. 2014; Hildebrand 2015). Genetic mutations that are associated with abnormalities in joint shape are also highly correlated with increased susceptibility to OA development (Baker‐Lepain & Lane 2010; Waarsing *et al*. 2011). Other genetic predisposition to OA development has been described (Tsezou 2014; Reynard 2017; Wang *et al*. 2016) but it remains largely unknown, however, whether these influence initiation and/or progression specifically.

Although trauma and injury may be the initiating factor leading to OA initiation in some groups, the development of OA is most likely due to the interaction between multiple risk factors. Further knowledge of how these interact to initiate disease may help differentiate between other patient subsets and may allow for more targeted therapy.

## Separating initiation and progression phases using human tissues

Human samples are difficult to obtain, especially at different stages of disease. Samples can be obtained following total or partial joint replacement for severe/late OA, and controls normally from amputations from other non‐arthritic causes (such as trauma, necrosis from severe diabetes). But this has led to research concentrating on late disease because firstly it is difficult to define early OA clinically as there is currently a lack of effective early disease markers and secondly collecting cartilage from patients with early‐stage OA is highly invasive and may lead to an acceleration in disease progression. Some researchers have countered this issue using tissue from patients with osteoarthritis from different locations of the joint; indeed, these studies assumed that the tissue that is the closest to the lesion represents the most advanced OA, and thus, the further away from the lesion, the more ‘normal’ the tissue. Therefore, a gradation in disease severity in the same joint is used to determine the changes in cartilage and chondrocyte behaviour with disease advancement. This does not take into account that the joint environment in these OA joints may still be highly abnormal; thus, even ‘normal’‐looking tissue may still be compromised; for example, OA synovium has a very different composition, including high levels of degrading enzymes and cytokine levels (Struglics *et al*. 2015; Bigoni *et al*. 2016; Liu *et al*. 2016), which would affect the chondrocyte responses and expression profiles.

The use of such protocols has identified various pathways and molecular markers for early and late OA. Indeed, comparison between damaged and undamaged areas of the condyle, as described in (Snelling *et al*. 2014), shows expression patterns of genes involved in cell signalling, extracellular matrix remodelling and inflammatory responses; these include matrix metalloproteinases (MMPs), growth factor signalling proteins, collagens and SOX 9, amongst other common OA genes (Sato *et al*. 2006; Geyer *et al*. 2009). Fukui *et al*. (2008) have separated the severity of degradation by grouping the samples (from the same OA patients’ joint) into four distinct areas depending on the zones of the articular cartilage affected (preserved area, damaged with superficial, middle and deep zones present, damaged with middle and deep zones, damaged with deep zones only). They found that the profile of gene expression differed dependently on the zones and the extent of cartilage degeneration.

Other studies have analysed synovial fluid or cartilage tissue from normal compared with early and advanced OA. Heard *et al*. (2012) used criteria for the severity of disease based on arthroscopic examination and X‐ray analysis. Unfortunately, this study did not find any significant markers for early OA. Another study determined early OA as patients undergoing surgery for meniscal tears (during which surgery mild cartilage degeneration was observed), whereas late OA as patients undergoing total joint replacement (Ritter *et al*. 2013). This study found almost identical gene expression patterns between early OA and late OA compared with healthy controls. These and other similar studies (Gobezie *et al*. 2007) suggest that these criteria for early and late OA may represent the same phase of disease in progression, and are too late to identify initiation events.

In an attempt to identify markers of early and late diseases, one study used cartilage from sarcomas in lower extremities of patients and separated them into early‐, with signs of some fibrillation upon visual examination, and normal‐, as well as late‐stage OA following total knee replacement (Lorenzo *et al*. 2004). Proteomic analysis was performed on these tissues and showed that newly synthetized cartilage proteins were altered in a similar manner in early and late OA, including increased expression of cartilage oligomeric matrix protein (COMP), fibronectin and cartilage intermediate layer protein (CILP). This further suggests that early OA and late OA follow a common molecular pathway. These potential markers of disease were also confirmed in a mouse model with increased serum levels of COMP with onset of cartilage degeneration (Salminen *et al*. 2000).

## Using time in well‐described animal models

Animal models are a great source of information to determine the sequence of events that take place during OA development in response to known initiating factors. Whilst model systems have been developed in a number of species, mice are especially widely used due to the abundant genetically modified strains available.

### The Str/ort mouse shows progressive molecular changes with OA comparable to human disease

A well‐described model of spontaneous OA is the Str/ort mouse (Mason *et al*. 2001). This model shows similar patterns of disease as those described in human OA, including proteoglycan loss, AC degradation by A disintegrin and metalloproteinase with thrombospondin motifs (ADAMTS4‐5, major aggrecanases) and MMPs, and subchondral bone sclerosis. Disease development in this model has been well described, with 8‐week‐old joints showing no apparent disease, lesions appeared from 18 weeks of age and progressed further with age. This timing was used in a microarray study in which RNA gene expression in AC was assessed in 8 weeks (before apparent disease), 18 weeks (early signs of AC degradation) and 40 weeks of age (severe disease (Poulet *et al*. 2012)). Gene expression analysis showed 115 differentially regulated genes between 8 and 18 weeks, including genes involved in matrix synthesis and degradation, which could represent the switch between initiation and progression of AC degradation in OA. Interestingly, no changes were seen between early and late diseases, as seen in human studies mentioned above. Analysis of genes expression between Str/ort and CBA control mice at 8 weeks found that genes linked to the NFkB signalling pathway may be involved in initiation of disease in this model.

### The surgical DMM model is the main post‐traumatic OA model in mouse studies

The most widely used model of OA in the mouse is the surgical model DMM (destabilization of the medial meniscus). In this case, the initiating factor is mechanical and starts with the destabilization of the joint. Changes as early as 6 hours following surgery, which included cartilage matrix proteases, were thought to be due to abnormal mechanical input induced in this model (Burleigh *et al*. 2012). Studies have used inhibitors to decrease disease severity showing potential for these targets as preventative and/or to slow progression; indeed, increasing the expression of PRG4 either before or after injury resulted with the same protection, suggesting the main effect is achieved during progression and not initiation (Ruan *et al*. 2013). Thus, this target could be used in later stages of OA. Similarly, Li *et al*. (2015) used resveratrol treatment beginning at 4 weeks after DMM surgery and still found a beneficial effect with a preservation of the AC and subchondral bone structural homeostasis, suggesting an effect on progression.

## Reversible versus irreversible stages

### Cartilage degradation reversibility as a hallmark of OA progression?

A main hallmark of OA is the active degradation of the extracellular matrix of AC, in particular of the two main components of this tissue, namely collagen type II and aggrecan. A molecular model of AC degradation reversibility has been put forward based on both *in vitro* and *in vivo* experiments. This model suggests that ADAMTS‐mediated aggrecan degradation was reversible, whereas MMP‐mediated aggrecan and collagen type II degradation was irreversible (van Meurs *et al*. 1999; Karsdal *et al*. 2008; Bay‐Jensen *et al*. 2010). This suggests that a switch in ADAMTS to MMP activity may be a marker of progression over initiation, by crossing a ‘point‐of‐no‐return’ discussed in a review by Bay‐Jensen *et al*. (2010). He suggests that this event of irreversibility may involve the change in phenotype of the chondrocyte from a ‘normal’ phenotype to a catabolic, hypertrophic and/or dedifferentiated phenotype. In this scenario, the chondrocyte is unable to maintain a healthy AC matrix. A marker of hypertrophic chondrocytes is the synthesis of MMP13, thus implying the possibility that chondrocyte phenotype change and MMP‐mediated degradation are linked.

### Reversible changes in non‐cartilaginous tissue may be apparent from the transient nature of their changes

It is also important to remember that OA is a disease involving various tissues in the joint. Synovial fibrosis and hyperplasia have been shown to be transient in a murine model of non‐invasive trauma (Poulet *et al*. 2011) and in response to connective tissue growth factor treatment *in vivo* (Blaney Davidson *et al*. 2006), but fibrosis becomes permanent in response to TGFβ treatment. Thus, TGFβ activity in the synovium may represent a switch to irreversibility for OA‐related synovial changes. In addition, epiphyseal and subchondral bone changes can be reversed upon treatment in human and animal studies (Lv *et al*. 2014; Cui *et al*. 2015; Miller *et al*. 2015). Subchondral bone thickening with non‐repetitive regimes of mechanical stimulation in mice are transient changes, as opposed to bone changes induced by repetitive regimes (Poulet *et al*. 2015).

This suggests that the ‘point‐of‐no‐return’ that separates initiation and progression may be linked to chondrocyte hypertrophy, with increases in MMP13 expression and MMP‐mediated cartilage degradation, and with TGFβ activity.

## Initiation does not necessarily mean progression

In the human population, a wide range of individuals show signs of cartilage defects with no other evidence of OA‐like pathology. Indeed, one small study reports that amongst 13 normal healthy individuals, nine had signs of focal cartilage abnormalities (Stahl *et al*. 2009). Another slightly bigger study (with 297 subjects, all with no significant current or past knee disease) found that 62% of the subjects had tibiofemoral cartilage defects (Racunica *et al*. 2007). Professional athletes are also prone to chondral defects, with 36% being affected in another study, whereas 14% of these same athletes were asymptomatic (Flanigan *et al*. 2010).

Animal models of trauma have also shown that articular cartilage lesions do not necessarily progress into osteoarthritis. Indeed, a single non‐invasive mechanical trauma protocol induced localized articular cartilage lesions, without ruptures or apparent tears in the ligaments or menisci, in the lateral femur of a CBA mouse knee joint (Poulet *et al*. 2011). Knees after this trauma regime were followed for 5 weeks, and up to 8 weeks, and did not show any worsening of the lesion severity. Thus, damage to the articulating surface alone, which cannot repair, is not sufficient to induce progressive OA degeneration.

## Separating initiation and progression following joint trauma

### A non‐invasive model of mouse knee trauma can distinguish between initiation and progression

Most animal models of OA used to date have allowed the definition of the main OA hallmarks and their mechanisms, and some were used to test possible candidates to slow disease progression. But most are not able to distinguish between initiation and progression. A recent model of mechanical trauma was able to induce reproducible AC lesions in the same mouse strain (CBA), which progress with time with a repetitive regime (Poulet *et al*. 2011). This same model was also used to induce AC lesions that do not worsen with time, using a single loading episode. Comparing these two regimes will allow us, for the first time, to define the mechanisms involved between initiation and progression. We have shown using this model that proteoglycan loss was one of these events that differentiate between progressive and non‐progressive lesions.

### The conjunction of a spontaneous model (Str/ort) and non‐invasive trauma model of OA can further inform us on initiation and progression

This model was also used to determine the effect of mechanical loading on initiation and progression. The Str/ort mouse develops spontaneous OA lesions primarily in the medial tibia. Thus, mechanical loading of young Str/ort mice, in the early phases of OA development, resulted in an acceleration in progression of spontaneous OA lesions in the medial tibia (Poulet *et al*. 2013). This was marked by increased proteoglycan loss and collagen type II degradation by MMPs. The lateral femur, which is targeted in the trauma model, is the least affected by spontaneous OA in the Str/ort mouse. Initiation of mechanical lesions following trauma was reduced in this mouse, suggesting that mechanical loading can have different effects on different phases of disease and that the Str/ort mouse does not develop OA because of a major deficit in AC mechanical properties. The study of the specific cellular processes involved in the acceleration of OA lesions in the medial tibia will allow defining new targets for slowing the rate of progression.

## Conclusions

Stratification of disease phases in osteoarthritis is necessary to define targets for therapy, as well as markers of disease severity. These will include the development of appropriate therapeutic strategies, such as prevention, blocking and slowing progression and repair, before the need for total joint replacement in end‐stage disease. The model of non‐invasive trauma described above (Poulet *et al*. 2011) allows for the distinction between initiation and progression to be made: comparing joints loaded with a single loading episode or repetitive regimes will permit to determine factors involved between initiation and progression phases. In other models, involving specific interventions to induce OA (such as surgery), the effects of specific targets or mechanisms on initiation and progression, respectively, can be achieved with different timings of treatment or gene deletions such as before and after surgery. This distinction becomes more challenging when using chronic models such as ageing‐associated Str/ort mouse: in this model, its well‐described timing of pathology can give clues as to which stage the disease might be at, and represents a good testing group for primary human OA. In the long term, this will further determine the effectiveness of the target in the each phase of OA for therapy as well as a marker of disease, leading to more patient‐specific time‐dependent treatments.

## Conflict of interest

The author has no conflict of interest.

## Funding source

Dr Poulet is funded by Arthritis Research UK (20859).
